# Unilateral bulge after dermatomal rash

**DOI:** 10.1016/j.jdcr.2024.11.024

**Published:** 2024-11-30

**Authors:** Timothy Blackwell, Allen Sapadin

**Affiliations:** Dermatology Department, Hackensack University Medical Center-Palisades, North Bergen, New Jersey

**Keywords:** dermatomal rash, herpes zoster, pseudohernia, unilateral bulge, VZV

## Case

**Fig 1:**
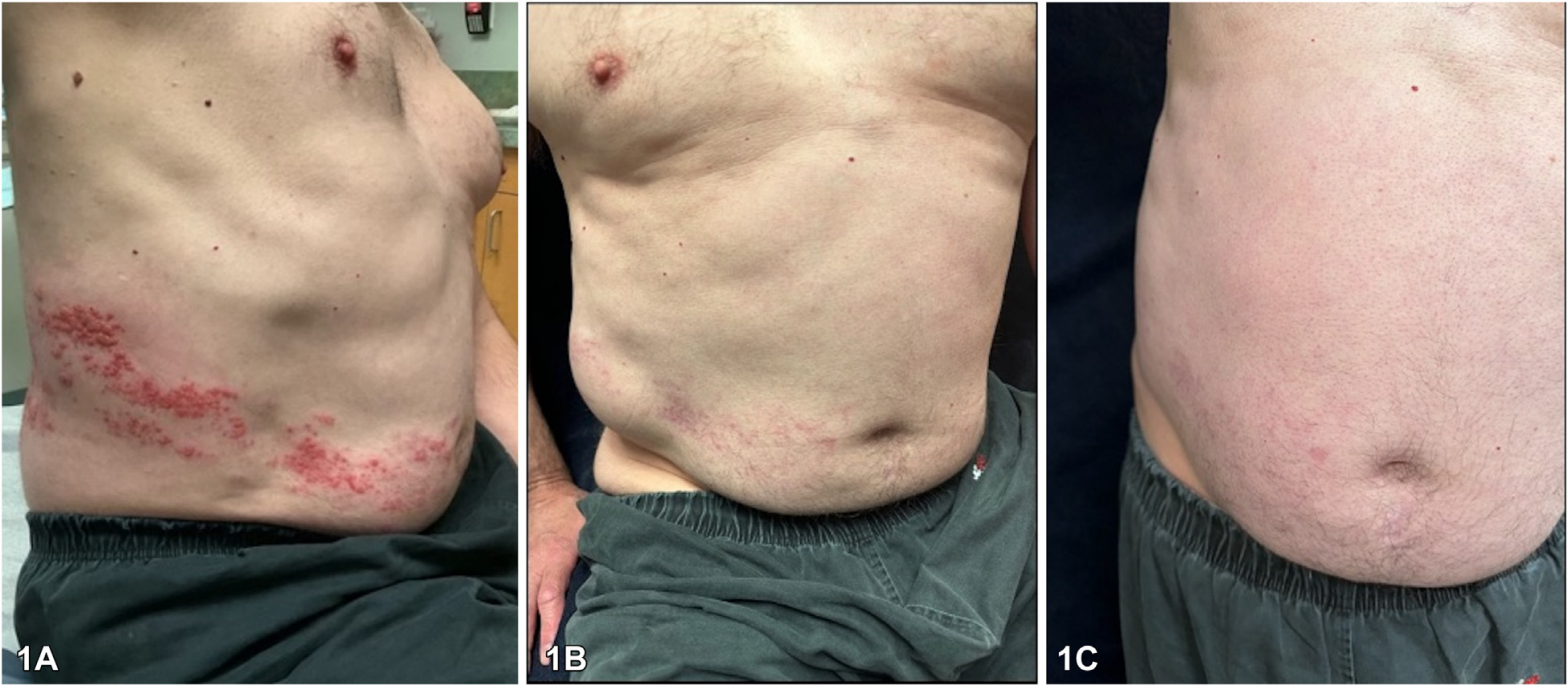

A 66-year-old male presented with a unilateral flank bulge (Fig 1) for the past 2 weeks in the area of a resolving dermatomal rash after taking valacyclovir 1g three times a day for 7 days. The patient is a martial arts expert. There was loss of sensation overlying the soft, nontender bulge but patient denied fevers, chills, or abdominal pain. Ultrasound was performed and did not show tumor or herniation. A computed tomography scan was ordered but the patient did not receive the test because the nodule was resolving over the next 4 weeks.

**Question 1: What is the most likely diagnosis?**

A. Hernia
B. Pseudohernia
C. Lipoma
D. Anetoderma
E. Hematoma

**Answers:**

A. Hernia – Incorrect. Hernia is the most dangerous entity to rule out in this patient which is the reason for images. Other bedside tests such as auscultation can be done.
B. Pseudohernia – Correct. This complication occurs in 3% to 5% of patients with herpes zoster with a mean time of onset of skin eruption to pseudohernia is 3.5 weeks. It occurs because the inflammation of the nerve is superimposed on a previous pre-existing condition involving a strain on the innervated abdominal musculature such as distention from constipation or strain from excessive abdominal exercises.^1^
C. Lipoma – Incorrect. While this is certainly in the differential, an ultrasound could be able to characterize this lesion and for a lipoma to appear so rapidly is very rare.
D. Anetoderma – Incorrect. This can present as an asymptomatic bulge after an inflammatory insult; however, these lesions do not resolve over time.
E. Hematoma – Incorrect. These subcutaneous nodules are often much smaller and classically painful. These lesions usually grow over a span of months, not weeks.

**Question 2: What is next best step?**

A. Continue valacyclovir
B. Reassurance
C. Surgical repair
D. Ibuprofen
E. Electrodiagnostic study

**Answers:**

A. Continue valacyclovir – Incorrect. This is incorrect because the patient’s herpes zoster was already treated correctly.
B. Reassurance – Correct. This lesion is self-limiting and confirming this diagnosis with imaging may be necessary. There is no treatment necessary but in rare cases, constipation, paralytic ileus, and gait disturbance has been reported.^2^
C. Surgical repair – Incorrect. This is a pseudohernia and there is not a true outpouching requiring surgical repair. This is also not a lipoma.
D. Ibuprofen – Incorrect. These lesions are painless and there is no reason to treat medically.
E. Electrodiagnostic study – Incorrect. This is the most sensitive diagnostic tool for postherpetic abdominal pseudohernia, but this patient’s symptoms were resolving so it is unnecessary to order additional tests.^3^

**Question 3: What is the mean time for resolution?**

A. 4 weeks
B. 2 months
C. 5 months
D. 12 months
E. 24 months

**Answers:**

A. 4 weeks – Incorrect. The mean latent period from rash to abdominal muscle weakness is 3.5 weeks.
B. 2 months – Incorrect. This is incorrect but this is about when our patient’s abdominal pseudohernia started to resolve.
C. 5 months – Correct. Complete recovery was seen in roughly 80% of patients with a mean recovery time of 4.9 months.^4^^,^^5^
D. 12 months – Incorrect. The majority of patients at 1 year would have had full resolution of symptoms.
E. 24 months – Incorrect. There are some cases that do not resolve. Physical therapy has been advocated as a treatment for cases that have not resolved within a year.

## Conflicts of interest

None disclosed.
